# High societal costs and reduced health-related quality of life in inflammatory and systemic immune disease-associated dilated cardiomyopathies

**DOI:** 10.1007/s11136-025-04027-5

**Published:** 2025-07-30

**Authors:** Maurits Sikking, Isabell Wiethoff, Michiel Henkens, Silvia Evers, Sophie Stroeks, Max Venner, Jerremy Weerts, Hans-Peter Brunner-la Rocca, Christian Knackstedt, Vanessa van Empel, Michelle Michels, Stephane Heymans, Mickaël Hiligsmann, Job Verdonschot

**Affiliations:** 1 Department of Cardiology, Cardiovascular Research Institute Maastricht (CARIM), Maastricht University Medical Centre, P. Debyelaan 25, Maastricht, 6229 HX The Netherlands; 2 Department of Internal Medicine, Maastricht University Medical Centre, Maastricht, The Netherlands; 3 Department of Health Services Research, Care and Public Health Research Institute (CAPHRI), Maastricht University, Maastricht, The Netherlands; 4 Department of Pathology, Maastricht University Medical Centre, Maastricht, The Netherlands; 5 Netherlands Heart Institute (NLHI), Utrecht, The Netherlands; 6 Trimbos Institute, Netherlands Institute of Mental Health and Addiction, Centre for Economic Evaluation and Machine Learning, Utrecht, The Netherlands; 7 European Reference Network for Rare, Low Prevalence and Complex Diseases of the Heart (ERN GUARD-Heart), Maastricht, The Netherlands; 8 Department of Cardiology, Thoraxcenter, Erasmus MC, Rotterdam, The Netherlands; 9 Department of Cardiovascular Research, University of Leuven, Leuven, Belgium; 10 Department of Clinical Genetics, Maastricht University Medical Centre, Maastricht, The Netherlands

**Keywords:** Dilated cardiomyopathy, Aetiology, Quality of life, Societal cost, Genetic testing, Immune diseases, Inflammation

## Abstract

**Background:**

Dilated cardiomyopathy (DCM) comes with an estimated societal cost of above €10,000 per patient per year and a lower quality of life (QoL). However, DCM represents a heterogeneous disease with different aetiologies that can be combined in subgroups. Insight in QoL and societal costs per DCM subgroup may guide resource allocation, research focus areas, and tailored patient management. We investigated subgroup-specific costs and QoL in patients with DCM.

**Methods:**

Patients with DCM (*n* = 550) were included, all phenotyped by endomyocardial biopsy and genetic testing besides routine cardiac evaluation. Patients were classified as chemotherapy-induced DCM (*n* = 27), genetic DCM (*n* = 91), inflammatory DCM (*n* = 53), systemic immune-mediated disease (SID)-associated DCM (*n* = 83), alcohol-induced DCM (*n* = 40), and idiopathic DCM (*n* = 280). QoL and societal costs were cross-sectionally evaluated using the EQ-5D-5 L and the iMTA medical and productivity cost questionnaires, sent at 8 [IQR 5; 12] years after cardiomyopathy diagnosis.

**Results:**

Compared to other subgroups, QoL was lower for inflammatory DCM (-0.053 [-0.106; -0.003], *p* = 0.04), SID-associated DCM (-0.078 [-0.126; -0.031], *p* < 0.01), and alcohol-induced DCM (-0.079 [-0.141; -0.017], *p* = 0.01). Societal costs were higher for patients with inflammatory DCM (19,197€ [13,703 − 27,211]; log-transformed change + 0.862 [0.103; 1.621], *p* = 0.03), and SID-associated DCM (19,197€ [8,494 − 25,623]; log-transformed change + 0.804 {0.104; 1.505], *p* = 0.03). Healthcare costs were highest for patients with inflammatory DCM (6,198€ [4,083 − 8,626], log-transformed change + 0.614 [0.039; 1.189] *p* = 0.049).

**Conclusion:**

Patients with DCM due to chronic myocarditis or a SID have lower QoL and higher societal costs due to higher healthcare costs, compared to patients with DCM and other etiologies. These findings may guide resource allocation, research focus areas, and tailored management such as patient education, financial counseling, and psychological support.

**Supplementary Information:**

The online version contains supplementary material available at 10.1007/s11136-025-04027-5.

## Key learning points

What is already known

What this study adds

Dilated cardiomyopathy comes with a staggering societal cost of > 10.000€ per patient per year while impacting quality of life.

Dilated cardiomyopathy is a heterogeneous disease with diverse aetiologies.

Understanding underlying the impact of each aetiology on costs and quality of life may further guide resource allocation and patient management.

Aetologies of dilated cardiomyopathy come with their own societal cost characteristics and quality of life impact.

Inflammatory, systemic immune disease-associated, and alcohol-induced dilated cardiomyopathy have a worse quality of life, while the former two come with higher societal costs.

These findings help us in tailoring patient management such as in patient education, financial counseling, and psychological support.

**Supplementary Information:**

The online version contains supplementary material available at 10.1007/s11136-025-04027-5.

## Introduction

Dilated cardiomyopathy (DCM) is a common cause of heart failure and heart transplantation worldwide with an approximate lifetime incidence of 0.4% [1]. DCM comes with an estimated societal cost of above €10,000 per patient per year and a lower quality of life (QoL) [2]. However, the aetiology of DCM determines its clinical course and long-term prognosis [3, 4], and therefore, aetiology-derived subgroups may come with specific societal costs and QoL. Understanding the impact of these subgroups on this burden of DCM, may help prioritize resource allocation, guide research priorities, and facilitate tailored management such as patient education, financial counselling, and (psychological) support programs.

The burden of a disease encompasses its prevalence, and prognostic, psychological, and economical impact on the patient and society. Societal costs constitute the total costs of a disease for society and therefore includes healthcare-related costs such as healthcare visits, hospitalizations, diagnostic examinations and therapeutic interventions (i.e., including medications), and non-healthcare-related costs (e.g., loss of paid work productivity). Non-healthcare-related costs are an important cost driver in heart failure [2, 5] and often invisible or missed by the eye of the healthcare team. Insight into the total societal costs of a disease as well as QoL may therefore enlighten healthcare providers and provide guidance for resource allocation, research priorities, and tailored disease management.

Cost and QoL differences between aetiology-derived subgroups of DCM are reasonably expected but not previously assessed. These subgroups include *genetic DCM*, characterized by pathogenic gene variants; *inflammatory* and *systemic immune disease (SID)-associated DCM*, associated with infections and autoimmunity; and toxin-induced DCM, such as *alcohol-* and *chemotherapy-induced DCM* [1]. Additionally, when the aetiology is unknown, the condition is classified as *idiopathic DCM* [1]. Inflammatory [6], alcohol-induced [7], and genetic DCM [8–10], have a high risk of hospitalization and mortality. Therefore, QoL and societal costs may be different at least in these subgroups.

This study investigates the societal costs and QoL per aetiology-derived subgroup of DCM, using an extensively phenotyped cohort of patients in which genetic testing and endomyocardial biopsy (EMB) were routinely performed.

## Methods

### Study design and population

Patients with nonischaemic, nonvalvular DCM were recruited from January 2004 to December 2021 as part of the Maastricht cardiomyopathy registry (mCMP-registry) [11]. This registry includes patients with DCM referred to the DCM outpatient clinic at the Maastricht University Medical Centre (MUMC+) [11]. Inclusion criteria for this study were: (i) age ≥ 16 years (i.e., the legal age limit to provide informed consent without parental supervision in The Netherlands); (ii) written informed consent; and (iii) DCM defined as left ventricle ejection fraction (LVEF) < 50% (i.e., less than 50% of blood is pumped out of the heart per heart beat) [12]. To collect cross-sectional data on the burden of disease among DCM patients across all stages of the disease, patients were invited to complete the EQ-5D-5 L [13], the institute for Medical Technology Assessment (iMTA) Medical Consumption Questionnaire (iMCQ) [14], and the iMTA Productivity Cost Questionnaire (iPCQ) [15], at a random duration since cardiomyopathy onset (median 8 [interquartile (IQR) 5;12] years). The study was conducted in accordance with the Declaration of Helsinki and was approved by the institutional Medical Ethics Committee of the Maastricht University Medical Center (METC azM/UM) on the 1st of July of 2021 with registration number METC 21 − 017.

### DCM subgroup classification

We categorized patients with DCM according to aetiology. Chemotherapy and alcohol are toxic aetiologies of DCM [10, 16–18]. Chemotherapy was defined as chemotherapeutic regimens for cancer. Alcohol was defined as at least 21 units of alcohol per week for males and 14 units of alcohol for females (0.8 gram of alcohol = 1 unit) [17]. One patient had cocaine as a potential aetiology of DCM but since he was an alcohol consumer as defined above; he was classified as alcohol-induced DCM. Endomyocardial biopsy-proven cardiac inflammation and systemic immune disease were defined as inflammatory DCM, and SID-associated DCM, respectively. Endomyocardial biopsy-proven cardiac inflammation was in accordance with the latest position statement by the European Society of Cardiology (ESC): at least 14 leukocytes per mm^2^ or 7 T-cells per mm^2^ [19]. For SID-associated DCM, the latest position statement of the ESC on myocardial involvement in SIDs was used [20]. Patients underwent genetic testing as described previously [21], including all robust DCM-associated genes. Only variants classified as pathogenic or likely pathogenic in one of these genes were included as a genetic aetiology of DCM. In our subgroup allocation, all patients with a specific aetiology were allocated to that aetiology-specific group. For example, all patients with a genetic aetiology were allocated to the genetic DCM subgroup. In case they also fit criteria for inflammatory DCM, they were also allocated to that group. This method fits recent insights into aetiological overlap between patients with DCM [17, 18, 22–24]. Hence, the sum of all aetiology-derived subgroups is larger than the total cohort size (24 patients were allocated to more than one subgroup).

### Data collection

QoL was assessed using the EQ-5D-5 L questionnaire, comprising questions concerning the patient’s current health status across five dimensions: mobility (e.g., ability to walk), self-care (e.g., ability to wash or dress), usual activities (e.g., ability to work, study, perform housework, spend time with family, or do leisure activities), pain or discomfort, and anxiety or depression [13]. The EQ-5D-5 L was recently suggested to be a representable instrument to assess quality of life in patients with hypertrophic cardiomyopathy [25]. Individual health profiles were generated from the patient’s responses, and these profiles were converted into utility scores using the Dutch valuation set [26]. Utility scores represent QoL in numeric values where zero is a health state equivalent to death and one indicates perfect health.

Cost data were collected across four distinct categories: [1] healthcare costs [2], patient and family costs [3], productivity losses related to paid work, and [4] other costs associated with unpaid activities such as voluntary work [27]. Medical consumption was assessed using the iMCQ, covering various healthcare services utilized by patients including visits to general practitioners, social workers, physical therapists, occupational therapists, speech therapists, dieticians, alternative medical practitioners (e.g., homeopathy, acupuncture), psychologists/psychiatrists, company physicians, home care provided by care organisations, hospitalisations, and medications. In-hospital medical care was documented via diagnosis treatment combination (DBC) codes stored in the patient’s electronic health record for billing purposes by hospitals to insurance companies [14, 28, 29]. Additionally, the iMCQ included questions regarding patient and family costs, such as costs associated with unpaid home care provided by family members (for patients unable to fully care for themselves) and travel expenses for hospital visits. All items in the iMCQ retrospectively measure resource utilization over the preceding three months. For both medical consumption and patient and family costs, average resource utilization was computed for the entire sample by assigning zero volumes to patients who reported not having utilized any healthcare services. Final costs were determined by multiplying healthcare services used by standard prices outlined in the costing guideline of the Dutch Healthcare Institute (i.e. “Zorginstituut Nederland”) [30]. For medications, conservative prices, specifically the lowest-priced option based on the defined daily dosage, were selected [31]. If health services were not listed in the costing guideline, prices were sourced from the Dutch Healthcare Authority (NZA).

Productivity losses attributable to paid work and other costs associated with unpaid activities such as voluntary work were assessed using the iPCQ [15]. Specifically, the iPCQ collected data on the patient’s capacity to engage in paid work (disability), the number of lost work hours due to illness (absenteeism), reduced productivity at work due to illness (presenteeism), and the number of lost hours of unpaid activities such as voluntary work over a recall period of four weeks. Lost work hours were multiplied by standard tariffs as specified in the costing guideline [30]. Productivity losses due to disability, long-term sick leave, and lost hours of unpaid activities such as voluntary work were calculated using the friction cost approach, as recommended by the Dutch guidelines [32]. This approach terminates the calculation after twelve weeks, representing the time required for an employer to replace a sick worker [30]. Final costs were extrapolated to one-year period and reported as costs per patient per year (PPPY). All reference prices were adjusted for inflation using the data from the Dutch Central Bureau for Statistics (CBS) and are expressed in 2022 Euros [33]. A complete list of reference prices and assumptions made in this study was added to Supplemental material S1-4.

### Statistical methods

Variables are displayed as frequencies (percentage), mean ± SD, or median (IQR) as appropriate. Normality was assessed visually by using Q-Q-plots and histograms. Comparisons between groups were performed by using chi-square tests, Fisher exact, ANOVA, or Kruskal-Wallis, as appropriate. Yearly costs were logarithmically transformed due to right-skewness. Multiple linear regression analyses were performed for utility and yearly DCM costs as dependent variables. Age, sex, New York Heart Association classification (NYHA; a clinically established measurement of dyspnea symptoms), and the time between cardiomyopathy diagnosis and questionnaire, were selected as the independent variables for multiple linear regression. For internal validation, regression coefficients of QoL and yearly costs were bootstrapped using 2,000 replications and 95% confidence intervals were calculated using the percentile method. The combination of linear regression with bootstrapping is accepted by Dutch guidelines [34]. For consistency, bootstrapped values with 95% confidence intervals were also used for the description of the mean QoL as assessed by the utility score and societal costs per DCM subtype. The level of significance was *p* < 0.05 and tests were 2-sided. All statistical analyses were performed using RStudio version 4.0.4.

## Results

### Study population

A total of 673 ambulant patients with DCM invited to participate in this study of which 550 patients (82% response rate) completed all questionnaires. There were no differences between patients that did or did not fill in questionnaires in regard to standard clinical parameters such as age, sex and LVEF, as previously described [2]. Patients were classified according to aetiology (Table 1). These aetiology-derived subgroups differ according to age, sex, and the timing of completing the questionnaire since the diagnosis of DCM (Table 1). The median time between initial diagnosis of DCM and completion of the questionnaire in the overall cohort was 8 [5; 12] years.

**Table 1:** Patient characteristics per dilated cardiomyopathy subtype

	Alcohol-induced DCM	Chemo-therapy-induced DCM	Genetic DCM	Inflamma-tory DCM	SID-associated DCM	Idio-pathic DCM	Total cohort	*p*-value
	*N* = 40	*N* = 27	*N* = 91	*N* = 53	*N* = 83	*N* = 280	*N* = 550	
**Demographics**								
Age (years)	64 ± 11	62 ± 11	59 ± 11	61 ± 12	64 ± 11	61 ± 12	61 ± 12	**0.04**
Female	7 (18%)	19 (70%)	28 (31%)	20 (38%)	34 (41%)	93 (33%)	189 (34%)	**< 0.01**
Atrial fibrillation	9 (23%)	4 (15%)	24 (26%)	9 (17%)	20 (24%)	56 (20%)	118 (22%)	0.64
Hyper-tension	16 (40%)	4 (15%)	22 (24%)	12 (23%)	21 (25%)	88 (31%)	160 (29%)	0.14
COPD or asthma	4 (10%)	4 (15%)	11 (12%)	9 (17%)	16 (19%)	27 (10%)	66 (12%)	0.22
OSA	9 (23%)	5 (19%)	9 (10%)	11 (21%)	16 (19%)	36 (13%)	80 (15%)	0.19
Diabetes type 2	4 (10%)	5 (19%)	7 (8%)	4 (8%)	12 (15%)	21 (8%)	50 (9%)	0.24
BMI, kg/m^2^	27 ± 4	26 ± 5	27 ± 5	26 ± 4	27 ± 6	27 ± 5	27 ± 5	0.97
NYHA ≥III	10 (25%)	5 (19%)	17 (19%)	13 (25%)	20 (24%)	38 (14%)	94 (17%)	0.12
LVEF, %	46 ± 11	48 ± 10	44 ± 10	45 ± 13	45 ± 12	47 ± 11	46 ± 11	0.14
NT-proBNP, pmol/L	67 [29;193]	46 [13;154]	30 [9;55]	60 [13;103]	51 [14;97]	19 [7; 67]	31 [11; 79]	0.09
Time to question-naire, years*	6 [4;12]	7 [5;8]	9 [5;16]	11 [8;13]	9 [5;13]	7 [5;12]	8 [5;12]	**< 0.01**
**Cardiac devices**								
CRT	6 (15%)	2 (7%)	7 (8%)	6 (11%)	10 (12%)	45 (16%)	77 (14%)	0.35
ICD	4 (10%)	2 (7%)	26 (29%)	12 (23%)	18 (22%)	58 (21%)	112 (20%)	0.10
Pacemaker	1 (3%)	2 (7%)	1 (1%)	3 (6%)	0 (0%)	8 (3%)	15 (3%)	0.19

Ordinal variables are in numbers (percent). Normal distributed continuous variables are in mean ± standard deviation and non-normal distributed continuous variables are in median[interquartile range].

Abbreviations: DCM = dilated cardiomyopathy; SID = systemic immune disease; COPD = chronic obstructive pulmonary disease; OSA = obstructive sleep apnea; BMI = body-mass index; NYHA = New York Heart Association dyspnea classification; LVEF = left ventricle ejection fraction; CRT = cardiac resynchronization therapy; ICD = implantable cardioverter-defibrillator. *Time from cardiomyopathy diagnosis to questionnaire

### Comparison of quality of life per dilated cardiomyopathy subtype

**Fig. 1:**
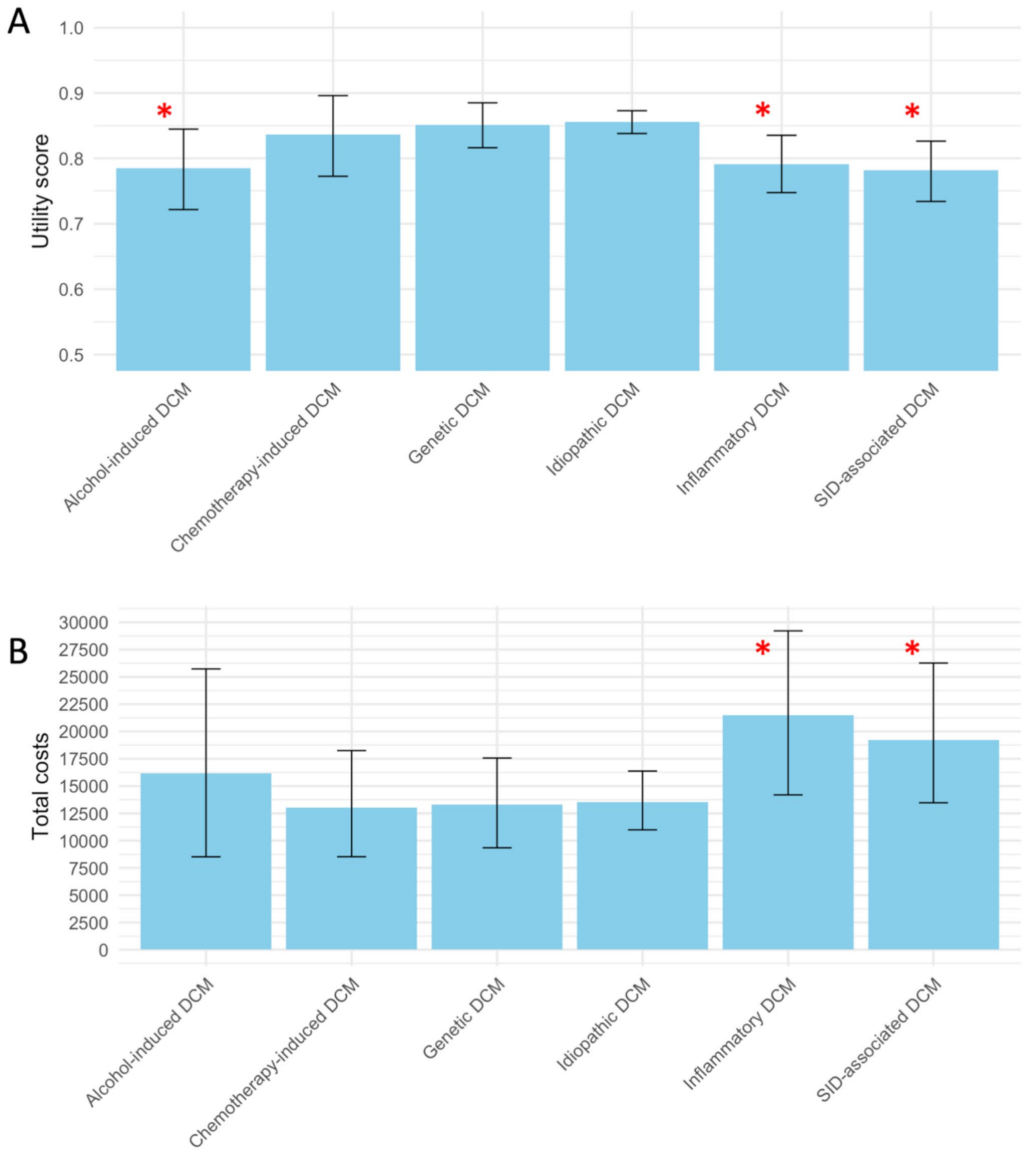
Comparison of quality of life and societal costs per dilated cardiomyopathy subtype *Asterisk: *p* < 0.05 in multivariate linear regression model including all other DCM subgroups as well as age, sex, New York Heart Association dyspnea classification and time from cardiomyopathy diagnosis to questionnaire

The mean values of the QoL as assessed by the utility score are reported in Table 2 and visualized in Fig. 1. QoL was lower in patients with inflammatory DCM (-0.054 [95% confidence interval (95%-CI) -0.106; -0.003], *p* = 0.04), SID-associated DCM (-0.078 [-0.126; -0.031], *p* < 0.01), and alcohol-induced DCM (-0.079 [-0.141; -0.017], *p* = 0.01), compared to patients with DCM in the other respective subgroups, independent of age, sex, NYHA class, and timing of questionnaire completion (Fig. 1; Table 3; Supplemental material S5).

**Table 2:** Quality of life and societal costs per aetiology-derived subgroup

Subtype	Quality of life, mean (95% CI)	Societal costs, mean (95% CI) pppy, in euros
Total cohort	0.840 (0.825–0.853)	14,814 (12,888 − 16,935)
Chemotherapy-induced DCM	0.837 (0.768–0.899)	13,023 (8,054 − 18,316)
Genetic DCM	0.851 (0.813–0.885)	13,288 (9,307 − 17,689)
Inflammatory DCM	0.792 (0.748–0.835)	21,443 (14,551 − 30,167)
SID-associated DCM	0.783 (0.734–0.829)	19,197 (13,703 − 27,211)
Alcohol-induced DCM	0.786 (0.721–0.846)	16,040 (8,494 − 25,623)
Idiopathic DCM	0.855 (0.837–0.873)	13,532 (10,995 − 16,318)

*Values represent bootstrapped means with 2000 replications.*

Abbreviations: pppy = per patient per year; DCM = dilated cardiomyopathy; SID = systemic immune disease; 95%CI = 95% confidence intervals

**Table 3:** Multivariate linear regression analysis of quality of life and societal costs according to dilated cardiomyopathy subtype

	QoL		Societal costs	
	Absolute change [95% CI]	*P*-value	Log-transformed change [95% CI]	*P*-value
Chemotherapy-induced DCM	-0.01 [-0.089; 0.051]	0.59	0.390 [-0.637; 1.418]	0.46
Genetic DCM	-0.019 [-0.070; 0.031]	0.45	0.140 [-0.608; 1.418]	0.71
Inflammatory DCM	-0.054 [-0.106; -0.003]	**0.04**	0.862 [0.103; 1.621]	**0.03**
SID-associated DCM	-0.078 [-0.126; -0.031]	**< 0.01**	0.804 [0.104; 1.505]	**0.03**
Alcohol-induced DCM	-0.079 [-0.141; -0.017]	**0.01**	0.645 [-0.262; 1.553]	0.16
Idiopathic DCM	-0.042 [-0.092; 0.009]	0.11	0.584 [-0.158; 1.326]	0.12
Age (per year)	-0.0003 [-0.0015; 0.0007]	0.49	-0.013 [-0.209; 0.557]	0.11
Female	-0.010 [-0.036; 0.017]	0.47	0.174 [-0.209; 0.557]	0.37
NYHA, ≥III	-0.244 [-0.275; -0.212]	**< 0.01**	1.638 [1.176; 2.101]	**< 0.01**
Time from cardiomyopathy diagnosis to questionnaire (per year)	0.0003 [-0.0008; 0.0014]	0.62	0.005 [-0.011; 0.021]	0.56
Intercept	0.953		8.194	
R^2^	0.346		0.116	

Each group category was compared to all other patients not fitting that group category

Abbreviations: DCM = dilated cardiomyopathy; SID = systemic immune disease; NYHA = New York Heart Association dyspnea classification; 95%CI = 95% confidence intervals

### Comparison of societal costs per dilated cardiomyopathy subtype

The mean societal costs PPPY are reported in Table 2 and visualized in Fig. 1. Societal costs were higher for patients with inflammatory and SID-associated DCM compared to the patients with DCM in the other subgroups, which was independent of age, sex, NYHA, and timing questionnaire completion (log-transformed cost increase of 0.862 (95%-CI 0.103–1.621), *p* = 0.03; and 0.804 (0.104–1.505), *p* = 0.03, respectively; Table 3; Supplemental material S5).

### Comparison of underlying cost factors per dilated cardiomyopathy subtype

**Fig. 2:**
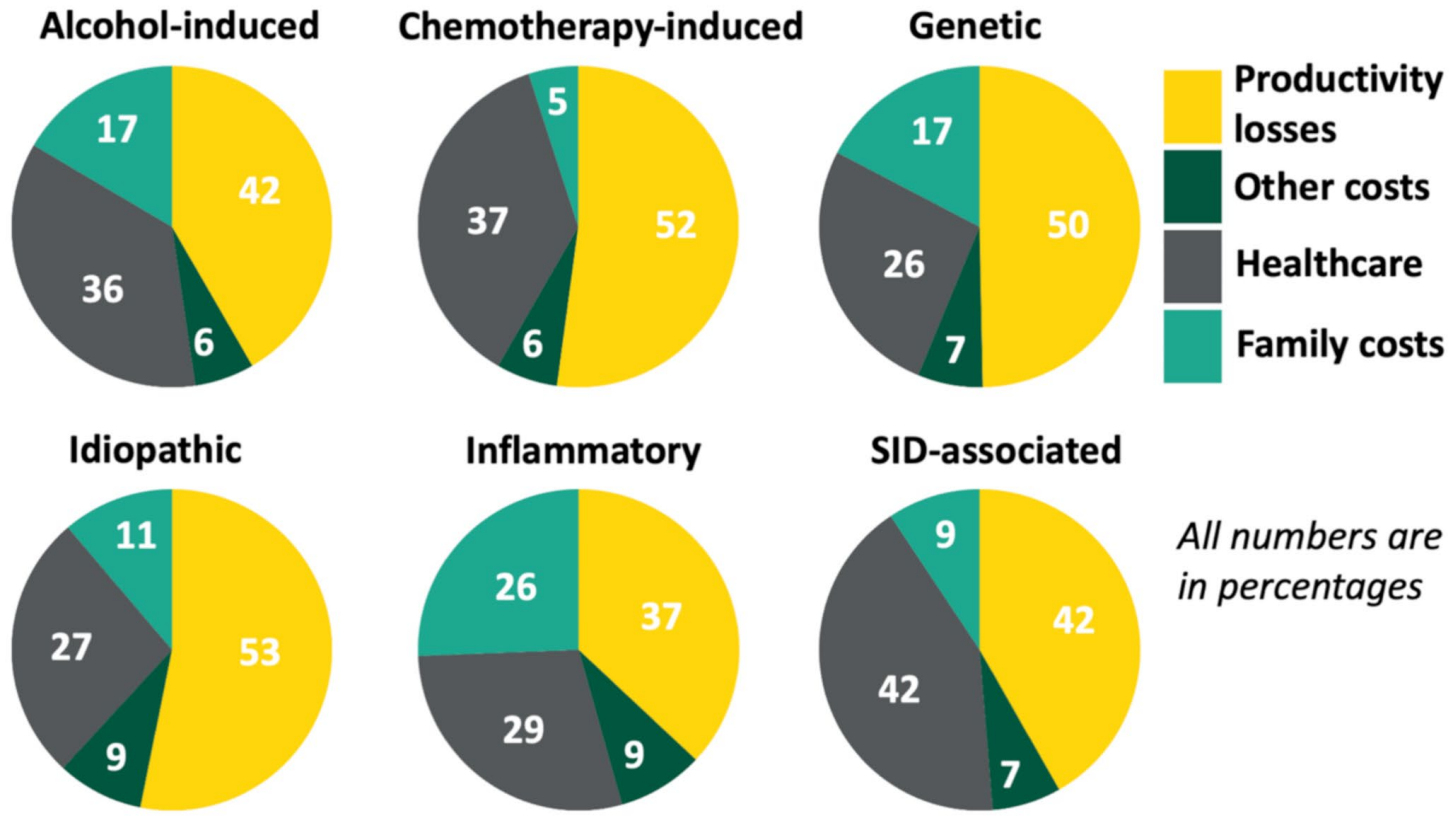
Cost distribution across dilated cardiomyopathy subgroups. Figure shows the distribution in percentages of each cost category underlying total societal cost calculation: healthcare-related costs (e.g., consultations, medication), family-related costs (e.g., unpaid home care provided by family members), productivity losses (e.g., lower productivity at paid jobs due to disability), and other costs (e.g., lower productivity at voluntary jobs)

The total societal cost can be divided into four categories: healthcare costs, family costs, productivity losses, and other costs (Fig. 2). The healthcare costs significantly differed among the subgroups, with a mean cost PPPY of €4,761 (95%-CI 8,054 − 18,316) for chemotherapy-induced DCM; €3,517 (2,769-4,417) for genetic DCM; €6,198 (4,083 − 8,626) for inflammatory DCM; €7,929 (4,313 − 14,086) for SID-associated DCM; €5,761 (2,883-9,964) for alcohol-induced DCM; and €3,637 (3,001–4,353) for idiopathic DCM (Fig. 2; Supplemental material S6). Healthcare costs PPPY were higher for inflammatory DCM independent of baseline differences (log-transformed cost increase of 0.712 (95%-CI 0.006–1.418), *p* = 0.049; Fig. 2 and Supplemental material S7).

As patients with inflammatory DCM are more likely to use (expensive) immunomodulatory therapies, we compared the medication costs as a possible explanation of the differences. There was no significant increase in medication costs between patients with inflammatory DCM compared to all other patients (log-transformed cost increase of -0.243 (95%-CI -0.811; 0.324), *p* = 0.40). Instead, the healthcare costs were mainly increased in patients with inflammatory DCM due to an increase in medical costs not related to medication, which is a combination of primary, home, outpatient, emergency, and inpatient care; log-transformed cost increase of 0.614 (95%-CI 0.039; 1.189), *p* = 0.04). The family costs, productivity losses, and other costs PPPY did not differ among DCM subgroups (Supplemental material S8-13).

## Discussion

DCM is a heterogeneous disease that can be divided into aetiology-derived subgroups by extensive phenotyping. This study reveals that these subgroups are associated with differences in QoL and societal costs. In particular, patients with alcohol-induced DCM, SID-associated DCM and inflammatory DCM experience a worse QoL, and the latter two also incur higher societal costs related to healthcare. These findings may guide resource allocation, research focus areas, and tailored management such as patient education, financial counseling, and psychological support.

### Differences in societal costs among subgroups of patients with DCM

Heart failure is progressively consuming a larger portion of healthcare budgets worldwide [5, 35]. However, previous estimates of the societal costs are mostly based on health care-related costs [5]. We previously estimated that the true economic burden may be more than twice as high in DCM mostly due to work productivity losses stressing the need to preserve or restore work ability in this patient population [2]. The current holistic study shows that specifically inflammatory and SID-associated subgroups of DCM incur high total societal costs, mainly due to healthcare costs not related to medication as medical costs were mainly related to contact with health professionals, diagnostic examinations, and interventions other than medication. As inflammatory and SID-associated DCM are less well-recognized and more difficult to diagnose compared to genetic or toxic-related DCM, a diagnostic delay might explain the more frequent need for health care. Future studies with larger numbers of these aetiologies could investigate differences in costs within the inflammatory DCM subgroup stratified on time to diagnosis. Previous studies investigating societal cost related to DCM did not report on the differences per subgroup, although this approach may be necessary to advance to more personalized health care [36–39].

### Differences in quality of life among subgroups of patients with DCM

QoL is often included as an important outcome parameter in clinical studies and is an independent predictor of poor outcome and disease progression in patients with heart failure [40]. Additionally, QoL correlates with the severity of symptoms and cardiac function in patients with DCM [2]. Within this disease, we observe a significant lower QoL in patients with inflammatory, SID-associated, and alcohol-induced DCM, compared to other forms of DCM. The new ESC guidelines for the management of cardiomyopathies prominently highlights the role of psychological care [41]. Our findings might point out that specific subgroups may benefit from proactively offering psychological care as our study suggests patients with inflammatory, SID-associated, and alcohol-induced DCM have a lower QoL compared to other patients with DCM. Interestingly, we did not find a significant lower QoL in genetic DCM although this form of DCM is associated with a worse clinical outcome [42]. Previous research investigating QoL in 60 patients with DCM also did not find a reduced QoL in patients with familial DCM (i.e., potentially genetic DCM) compared to patients without familial DCM [43]. The underlying reason for patients with genetic DCM to have a comparable QoL as patients with idiopathic DCM warrants further investigation. Speculatively, patients with inflammatory, SID-associated and alcohol DCM are yet underrecognized in the clinic. These findings highlight the importance of phenotyping and diagnostics in DCM, with potential impact of timely treatment to prevent a greater decrease in QoL, especially in patients with SID-associated and inflammatory DCM.

### Strengths and limitations

A strength of this study is the thorough investigation of the economic burden of DCM, in-depth clinical characterization of patients including endomyocardial biopsy and cardiogenetic testing, the novelty to assess QoL and societal costs in each aetiology-derived subgroup of DCM, and the inclusion of cost types beyond health care-related costs. Additionally, there was a long time between the diagnosis of DCM and the completion of the questionnaire, therefore we received information past the diagnostic process where patients are in routine follow-up. Limitations of this study include the lack of a validation cohort, the overlap between DCM subgroups, and the requirement to log-transform costs for regression analyses which complicates the interpretation of the findings. Costly and complex immune-related therapy regimes may have been underestimated in our calculations, especially in case inpatient care is required for injection. Although multiple patients had more than one aetiology, the number of included patients was too low to investigate the results with respect to multi-aetiology patients.

## Conclusion

The QoL and societal cost burden significantly differs among aetiology-derived subgroups of patients with DCM. Patients with inflammatory and SID-associated DCM have a lower QoL and associate with higher societal costs compared to other DCM subgroups mostly due to increased healthcare costs. These findings may guide resource allocation, research focus areas, and tailored management such as patient education, financial counseling, and psychological support.

## Electronic supplementary material

Below is the link to the electronic supplementary material.

Supplementary Material 1

## Data Availability

The data underlying this article will be shared on reasonable request to the corresponding author.

## Declarations

### Total word count

4563/5000 words.

### Corresponding author

Drs. M.A. Sikking, Department of Cardiology, Maastricht University Medical Center, P. Debyelaan 25, 6229 HX Maastricht, the Netherlands.
