# Supplementary material for: High societal costs and reduced health-related quality of life in inflammatory and systemic immune disease-associated dilated cardiomyopathies
Source: Qual Life Res. 2025 Jul 30;34(10):2901–10. doi: 10.1007/s11136-025-04027-5 (PMC12535485; doi:10.1007/s11136-025-04027-5)
Supplement: Supplementary file 1 — Supplementary Material 1 [file 11136_2025_4027_MOESM1_ESM.docx]

**Supplemental material**

- **Supplemental material S1. Overview reference prices and assumptions for healthcare costs**
- **Supplemental material S2. Overview reference prices and assumptions for family costs**
- **Supplemental material S3. Overview reference prices and assumptions for productivity losses**
- **Supplemental material S4. – Overview reference prices and assumptions for other costs**
- **Supplemental material S5. Bootstrapped multivariable regression of quality of life and societal costs.**
- **Supplemental material S6 Underlying healthcare costs per dilated cardiomyopathy subtype**
- **Supplemental material S7. Multivariate linear regression of healthcare costs per dilated cardiomyopathy subtype**
- **Supplemental material S8. Underlying family costs per dilated cardiomyopathy subtype**
- **Supplemental material S9. Multivariate linear regression of family costs per dilated cardiomyopathy subtype**
- **Supplemental material S10. Underlying productivity losses per dilated cardiomyopathy subtype**
- **Supplemental material S11. Multivariate linear regression of productivity losses per dilated cardiomyopathy subtype**
- **Supplemental material S12. Underlying other costs per dilated cardiomyopathy subtype**
- **Supplemental material S13. Multivariate linear regression of other costs per dilated cardiomyopathy subtype**

**Supplemental material S1. Overview reference prices and assumptions for healthcare costs**

CBS data used to transfer 2014 Euros into 2022 Euros: inflation factor = 1.221737084

| **Healthcare costs (iMCQ)** | | **Reference price** | **2022 costs** | **Source** |
| --- | --- | --- | --- | --- |
| **Outpatient care** | General Practitioner | 33 € | 40.32 € | Dutch Costing tool |
|  | Practice assistant (POH) | 17 € | 20.77 € | Dutch Costing tool |
|  | Social worker | 65 € | 79.41 € | Dutch Costing tool |
|  | Physiotherapist | 33 € | 40.32 € | Dutch Costing tool |
|  | Occupational therapist | 33 € | 40.32 € | Dutch Costing tool |
|  | Speech therapist | 30 € | 36.65 € | Dutch Costing tool |
|  | Dietician | 33 € | 40.32 € | Assumption |
|  | Homeopath or acupuncturist | 33 € | 40.32 € | Assumption |
|  | Psychologist | 94 € | 114.84 € | Dutch Costing tool |
|  | Company physician | 33 € | 40.32 € | Assumption |
| **Homecare** | Domestic support (per hour) | 23 € | 28.10 € | Dutch Costing tool |
|  | Self-care support (per hour) | 50 € | 61.09 € | Dutch Costing tool |
|  | Nursing (per hour) | 73 € | 89.19 € | Dutch Costing tool |
| **Medications** | Medication use (see Table 1.5) | Defined daily dosage price | - | Farmacotherapeutisch Kompas |
| **Emergency care** | First aid (EHBO) | 613 € | 748.92 € | Dutch Costing tool |
|  | Ambulance | 272 € | 332.31 € | Dutch Costing tool |
| **Specialist care** | Specialist care (average) | 91 € | 111.18 € | Dutch Costing tool |
| **Inpatient care** | Diagnostics & treatments performed at hospital  (see Table 1.6) | DBC code was translated into a “zorgproduct” code via the NZA website for which the respective tariff was selected | | NZA website ; Passantenprijslijst DBC & OZP 2022 - Maastricht UMC+ |
|  | Other diagnostics/treatments  - Residential care facility  - Rehabilitation care facility  - Psychiatric care facility  - Other care facility  - Sleep centre  (consultation)  - Pain clinic   (facial pain treatment)  - Self-help group  - Infusion centre  - Chiropractor  - Mitral varicose veins  - Maastricht Cardiology  centre (consultation) | 67 €  153 €  94 €  193.59 €  782 €  33 €  509.90 €  33 €  185.00 €  91 € | 81.86 €  186.93 €  114.84 €  193.59 €  782 €  40.32 € 509.90 €  40.32 €  185.00 €  111.18 € | Dutch Costing tool  Dutch Costing tool  Dutch Costing tool  Assumption (Nederlands Slaap Instituut)  Assumption based on Passantentarieven  Assumption (per session)  Assumption based on NZA  Assumption (per session)  Assumption based on Passantentarieven  Assumption (same as specialist care cardiologist) |
|  | Hospitalization (average) | 467 € |  | Dutch Costing tool |
|  | Overnight stays at different care facility  - Residential care facility  - Rehabilitation care facility  - Psychiatric care facility  - Other care facility  - Sleep centre  (Polysomnography)  - Trauma centre  (Trauma therapy)  - Epilepsy centre   (24h EEG) | 168 €  460 €  302 €  821.67 €  302 €  841.24 € | 205.25 €  562.00 €  368.96 €  821.67 €  368.96 €  841.24 € | Dutch Costing tool  Dutch Costing tool  Dutch Costing tool  Assumption (Nederlands Slaap Instituut)  Assumption (same tariff as psychiatric care facility)  Assumption based on Passantentarieven |

**Abbreviations:** NZA = Nederlands Zorgautoriteit; EHBO = Eerste hulp bij ongelukken

**Supplemental material S2. Overview reference prices and assumptions for family costs**

| **Family costs (iMCQ)** | | **Reference price** | **2022 costs** | **Source** |
| --- | --- | --- | --- | --- |
| **Homecare done by family member** | Family costs for care  - Domestic support  (per hour)  - Self-care support   (per hour)  - Practical support  (per hour) | - 14.00 € per hour  - 14.00 € per hour  - 14.00 € per hour | - 17.10 € per hour  - 17.10 € per hour  - 17.10 € per hour | Dutch Costing tool  Dutch Costing tool  Dutch Costing tool |
| **Travel costs** | Travel time to hospital  - Not applicable  - By foot  - Bike  - Car  - Public transport  - Taxi  - Other | - 0 €  - 0.19 € per km  - 0.19 € per km  - 0.19 € per km + 3€ parking costs  - 0.19 € per km  - 2.95€ basic tariff + 2.66 € per km  - 0.19 € per km | - 0 €  - 0.23 € per km  - 0.23 € per km  - 0.23 € per km +  3.67 € parking  - 0.23 € per km  - 3.60 € basis + 3.25 € per km  - 0.23 € per km | Assumption  Assumption  Dutch Costing tool  Dutch Costing tool  Dutch Costing tool  Assumption |

Underlying assumptions in the calculation:

- Costs calculated for the way towards and the way back from a hospital visit
- Patients going by foot, bike or in a different way have opportunity costs (time losses)

**Supplemental material S3. Overview reference prices and assumptions for productivity losses**

| **Productivity losses (iPCQ)** | **Reference price** | **2022 costs** | **Source** |
| --- | --- | --- | --- |
| Short-term productivity losses | - 34.75 € per hour | - 42.46 € per hour | Dutch Costing tool |
| Long-term productivity losses   - Friction cost method = replace sick worker after 12 weeks | - 34.75 € per hour | - 42.46 € per hour | Dutch Costing tool |
| Reduced productivity at work | - 34.75 € per hour | - 42.46 € per hour | Dutch Costing tool |

**Supplemental material S4. – Overview reference prices and assumptions for other costs**

| **Costs in other sectors (iPCQ)** | **Reference price** | **2022 costs** | **Source** |
| --- | --- | --- | --- |
| Unpaid work losses | - 14.00 € per hour | - 17.10 € per hour | Dutch Costing tool |

**Supplemental material S5. Bootstrapped multivariable regression of quality of life and societal costs.**

|  | Bootstrapped change in QoL  [95%CI] | Bootstrapped change in log-transformed societal costs  [95%CI] |
| --- | --- | --- |
| **Chemotherapy-induced DCM** | -0.019  [-0.093; 0.051] | 0.377  [-0.687; 1.442] |
| **Genetic DCM** | -0.018  [-0.075; 0.035] | 0.155  [-0.573; 0.932] |
| **Inflammatory DCM** | -0.052  [-0.108; -0.001] | 0.877  [0.163; 1.648] |
| **SID-associated DCM** | -0.077  [-0.132; -0.024] | 0.810  [0.171; 1.484] |
| **Alcohol-induced DCM** | -0.078  [-0.144; -0.019] | 0.669  [-0.242; 1.582] |
| **Idiopathic DCM** | -0.041  [-0.092; 0.008] | 0.593  [-0.151; 1.400] |
| **Age (per year)** | -0.000  [-0.001; 0.001] | -0.013  [-0.030; 0.005] |
| **Female** | -0.010  [-0.035; 0.016] | 0.181  [-0.203; 0.548] |
| **NYHA, ≥III** | -0.245  [-0.285; -0.206] | 1.642  [1.307; 1.996] |
| **Time from cardiomyopathy diagnosis to questionnaire (per year)** | -0.000  [-0.003; 0.001] | -0.000  [-0.040; 0.017] |
| Intercept | 0.954  [0.868; 1.036] | 8.22  [6.926; 9.537] |
| R^2^ | 0.360  [0.282; 0.436] | 0.133  [0.096; 1.760] |

Abbreviations: DCM = dilated cardiomyopathy; SID = systemic immune disease; NYHA = New York Heart Association dyspnea classification; 95%CI = 95% confidence intervals.

Significance codes: * <0.05; ** <0.01; *** <0.001.

**Supplemental material S6. Underlying healthcare costs per dilated cardiomyopathy subtype**

| Patient characteristics | | |
| --- | --- | --- |
| **DCM subtype** | **Cohort mean±SD** | **Bootstrapped 95% confidence interval** |
| **Total cohort** | 4,600±11,407 | 4,616  [3,808; 5,655] |
| **Chemotherapy-induced DCM** | 4,737±6,924 | 4,761  [2,554; 7,525] |
| **Genetic DCM** | 3,512±4,064 | 3,517  [2,769; 4,417] |
| **Inflammatory DCM** | 6,204±8,859 | 6,198  [4,083; 8,626] |
| **SID-associated DCM** | 8,093±24,857 | 7,929  [4,313; 14,086] |
| **Alcohol-induced DCM** | 5,776±12,003 | 5,761  [2,883; 9,964] |
| **Idiopathic DCM** | 3,638±5,587 | 3,637  [3,001; 4,353] |

**Supplemental material S7. Multivariate linear regression of healthcare costs per dilated cardiomyopathy subtype**

|  | Change in log-transformed healthcare costs  [95%CI] | Bootstrapped change in log-transformed healthcare costs  [95%CI] |
| --- | --- | --- |
| **Chemotherapy-induced DCM** | 0.357  [-0.599; 1.313] | 0.334  [-0.638; 1.299] |
| **Genetic DCM** | 0.349  [--0.346; 1.046] | 0.358  [-0.304; 1.104] |
| **Inflammatory DCM** | 0.712 *****  [0.006; 1.418] | 0.728  [0.085; 1.389] |
| **SID-associated DCM** | 0.504  [-0.148; 1.156] | 0.506  [-0.087; 1.121] |
| **Alcohol-induced DCM** | 0.664  [-0.181; 1.509] | 0.670  [-0.103; 1.522] |
| **Idiopathic DCM** | 0.525  [-0.165; 1.216] | 0.522  [-0.139; 1.294] |
| **Age (per year)** | 0.023 ******  [0.009; 0.038] | 0.024  [0.007; 0.041] |
| **Female** | 0.148  [-0.209; 0.505] | 0.153  [-0.201; 0.503] |
| **NYHA, ≥III** | 1.125 *******  [0.695; 1.556] | 1.136  [0.779; 1.497] |
| **Time from cardiomyopathy diagnosis to questionnaire (per year)** | 0.006  [-0.009; 0.021] | -0.000  [-0.037; 0.015] |
| Intercept | 5.144  [4.008; 6.279] | 5.17  [3.854; 6.462] |
| R^2^ | 0.082 | 0.101  [0.062; 0.151] |

Abbreviations: DCM = dilated cardiomyopathy; SID = systemic immune disease; NYHA = New York Heart Association dyspnea classification; 95%CI = 95% confidence intervals.

Significance codes: * <0.05; ** <0.01; *** <0.001.

**Supplemental material S8. Underlying family costs per dilated cardiomyopathy subtype**

| Patient characteristics | | |
| --- | --- | --- |
| **DCM subtype** | **Mean±SD** | **Bootstrapped 95% confidence interval** |
| **Total cohort** | 1,995±10,655 | 2,005  [1,212; 2,987] |
| **Chemotherapy-induced DCM** | 655±2,239 | 662  [107; 1,620] |
| **Genetic DCM** | 2,267±10,860 | 2,269  [535; 4,893] |
| **Inflammatory DCM** | 5,505±22,712 | 5,446  [732; 12,435] |
| **SID-associated DCM** | 1,797±5,675 | 1,772  [724; 3,086] |
| **Alcohol-induced DCM** | 2,640±8,709 | 2,625  [588; 5,642] |
| **Idiopathic DCM** | 1,525±9,735 | 1,496  [680; 2,839] |

**Supplemental material S9. Multivariate linear regression of family costs per dilated cardiomyopathy subtype**

|  | Change in log-transformed family costs  [95%CI | Bootstrapped change in log-transformed family costs  [95%CI] |
| --- | --- | --- |
| **Chemotherapy-induced DCM** | 0.813  [-0.762; 2.387] | 0.796  [-0.597; 2.212] |
| **Genetic DCM** | -0.338  [-1.48; 0.807] | -0.323  [-1.528; 0.898] |
| **Inflammatory DCM** | 0.376  [-0.787; 1.538] | 0.414  [-0.931; 1.731] |
| **SID-associated DCM** | 0.778  [-0.296; 1.851] | 0.783  [-0.299; 1.795] |
| **Alcohol-induced DCM** | 0.821  [-0.570; 2.211] | 0.867  [-0.605; 2.347] |
| **Idiopathic DCM** | 0.210  [-0.927; 1.347] | 0.225  [-1.011; 1.408] |
| **Age (per year)** | 0.012  [-0.012; 0.036] | 0.012  [-0.012; 0.035] |
| **Female** | 0.396  [-0.192; 0.983] | 0.422  [-0.175; 1.015] |
| **NYHA, ≥III** | 2.470 *******  [1.761; 3.178] | 2.471  [1.666; 3.301] |
| **Time from cardiomyopathy diagnosis to questionnaire (per year)** | 0.014  [-0.011; 0.039] | 0.009  [-0.035; 0.037] |
| Intercept | 1.060  [-0.809; 2.930] | 1.075  [-0.817; 2.933] |
| R^1^ | 0.110 | 0.128  [0.075; 0.189] |

Abbreviations: DCM = dilated cardiomyopathy; SID = systemic immune disease; NYHA = New York Heart Association dyspnea classification; 95%CI = 95% confidence intervals.

Significance codes: * <0.05; ** <0.01; *** <0.001.

**Supplemental material S10. Underlying productivity losses per dilated cardiomyopathy subtype**

| Patient characteristics | | |
| --- | --- | --- |
| **DCM subtype** | **Mean±SD** | **Bootstrapped 95% confidence interval** |
| **Total cohort** | 7,031±14,631 | 7,016  [5,821; 8,336] |
| **Chemotherapy-induced DCM** | 6,822±9,630 | 6,810  [3,320; 10,552] |
| **Genetic DCM** | 6,616±12,319 | 6,584  [4,409; 9,166] |
| **Inflammatory DCM** | 7,907±10,971 | 7,948  [5,169; 10,976] |
| **SID-associated DCM** | 8,016±15,505 | 7,953  [4,981; 11,559] |
| **Alcohol-induced DCM** | 6,750±17,944 | 6,836  [2,205; 12,925] |
| **Idiopathic DCM** | 7,197±15,993 | 7,205  [5,551; 9,121] |

**Supplemental material S11. Multivariate linear regression of productivity losses per dilated cardiomyopathy subtype**

|  | Change in log-transformed productivity losses  [95%CI] | Bootstrapped change in log-transformed productivity losses  [95% CI] |
| --- | --- | --- |
| **Chemotherapy-induced DCM** | 0.821  [-1.337; 2.979] | 0.800  [-1.537; 3.106] |
| **Genetic DCM** | 0.291  [-1.280; 1.861] | 0.318  [-1.228; 1.955] |
| **Inflammatory DCM** | 0.944  [-0.650; 2.538] | 0.953  [-0.761; 2.669] |
| **SID-associated DCM** | 0.886  [-0.586; 2.357] | 0.901  [-0.705; 2.488] |
| **Alcohol-induced DCM** | -0.260  [-2.166; 1.646] | -0.216  [-2.161; 1.702] |
| **Idiopathic DCM** | 0.526  [-1.032; 2.084] | 0.546  [-1.030; 2.083] |
| **Age (per year)** | -0.144  [-0.177; -0.111] | -0.144  [-0.178; -0.111] |
| **Female** | -0.389  [-1.194; 0.416] | -0.374  [-1.133; 0.382] |
| **NYHA, ≥III** | 2.430 ***  [1.459; 3.401] | 2.446  [1.465; 3.419] |
| **Time from cardiomyopathy diagnosis to questionnaire (per year)** | -0.006  [-0.040; 0.028] | -0.011  [-0.078; 0.034] |
| Intercept | 11.37  [8.809; 13.935] | 11.414  [8.643; 14.252] |
| R^1^ | 0.173 | 0.190  [0.136; 0.251] |

Abbreviations: DCM = dilated cardiomyopathy; SID = systemic immune disease; NYHA = New York Heart Association dyspnea classification; 95%CI = 95% confidence intervals.

Significance codes: * <0.05; ** <0.01; *** <0.001.

**Supplemental material S12. Underlying other costs per dilated cardiomyopathy subtype**

| Patient characteristics | | |
| --- | --- | --- |
| **DCM subtype** | **Mean±SD** | **Bootstrapped 95% confidence interval** |
| **Total cohort** | 1,179±4,091 | 1,178  [863; 1,544] |
| **Chemotherapy-induced DCM** | 806±1,781 | 796  [236; 1,467] |
| **Genetic DCM** | 883±3,894 | 888  [277; 1,842] |
| **Inflammatory DCM** | 1,878±4,382 | 1,866  [850; 3,158] |
| **SID-associated DCM** | 1,336±3,300 | 1,345  [723; 2,090] |
| **Alcohol-induced DCM** | 980±2,295 | 985  [346; 1,750] |
| **Idiopathic DCM** | 1,190±4,567 | 1,179  [705; 1,743] |

**Supplemental material S13. Multivariate linear regression of other costs per dilated cardiomyopathy subtype**

|  | Change in log-transformed other costs  [95%CI] | Bootstrapped change in log-transformed other costs  [95%CI] |
| --- | --- | --- |
| **Chemotherapy-induced DCM** | -0.221  [-1.871; 1.428] | -0.238  [-2.001; 1.533]] |
| **Genetic DCM** | -0.034  [-1.235; 1.167 | -0.043  [-1.235; 1.119] |
| **Inflammatory DCM** | 0.560  [-0.658; 1.778] | 0.542  [-0.756; 1.826] |
| **SID-associated DCM** | 0.607  [-0.518; 1.731] | 0.607  [-0.562; 1.773] |
| **Alcohol-induced DCM** | 0.040  [-1.417; 1.498] | 0.055  [-1.215; 1.426] |
| **Idiopathic DCM** | -0.131  [-1.323; 1.060] | -0.129  [-1.283; 1.005] |
| **Age (per year)** | -0.014  [-0.039; 0.011] | -0.014  [-0.038; 0.008] |
| **Female** | 0.447  [-0.168; 1.063] | 0.446  [-0.155; 1.039] |
| **NYHA, ≥III** | 2.352 *******  [1.610; 3.095] | 2.344  [1.415; 3.280] |
| **Time from cardiomyopathy diagnosis to questionnaire (per year)** | -0.003  [-0.029; 0.024] | 0.002  [-0.029; 0.056] |
| Intercept | 2.129  [0.170; 4.088] | 2.113  [0.254; 3.962] |
| R^1^ | 0.091 |  |

Abbreviations: DCM = dilated cardiomyopathy; SID = systemic immune disease; NYHA = New York Heart Association dyspnea classification; 95%CI = 95% confidence intervals.

Significance codes: * <0.05; ** <0.01; *** <0.001.
